# The use of individual, social, and animated cue information by capuchin monkeys and children in a touchscreen task

**DOI:** 10.1038/s41598-020-80221-4

**Published:** 2021-01-13

**Authors:** Elizabeth Renner, Donna Kean, Mark Atkinson, Christine A. Caldwell

**Affiliations:** 1 Psychology, Faculty of Natural Sciences, University of Stirling, Stirling, UK; 2 School of Management and School of Psychology and Neuroscience, University of St Andrews, St Andrews, UK; 3 Present Address: Department of Psychology, Durham University, Durham, UK

**Keywords:** Psychology, Human behaviour

## Abstract

The distinctiveness of human cumulative culture raises the question of whether humans respond differently to information originating from social sources, compared with information from other sources. Further, does any such differential responding set humans apart from other species? We studied how capuchin monkeys and 2- to 5-year-old children used information originating from their own actions, those of a human demonstrator, or an animated cue. This information, presented via a touchscreen, always revealed in the first trial (T1) the reward value (rewarded or unrewarded) of one stimulus from a 2- or 3-item array, and could be used in a follow-up trial (T2) involving the same stimulus array. Two monkeys achieved a level of proficiency indicating their appreciation of the T1–T2 relationship, i.e., reliably repeating rewarded (“win”) selections and actively avoiding repetition of unrewarded (“lose”) selections well above chance levels. Neither the two task-proficient monkeys nor the children showed overall performance differences between the three source conditions. Non-task-proficient monkeys, by contrast, did show effects of source, performing best with individually-acquired information. The overall pattern of results hints at an alternative perspective on evidence typically interpreted as showing a human advantage for social information use.

## Introduction

Human cultures are complex and elaborate, and influence many domains of behaviour. In comparison, animal cultures are generally restricted in complexity and domain. For example, cumulativity is a salient feature of human cultures, while thus far evidence indicates that it is either absent or more limited in non-human species^1–3^. The transmission of most cultural behaviours requires social learning. If humans use social information differently from non-human animals, this might explain the species difference in the qualities of cultural behaviours. To determine whether this might be the case, it is important to compare how humans respond to information from a social source (versus their own experience) and how non-humans respond to information from a social source (versus their own experience), and examine whether these groups show different patterns of information use. Generally, to compare the performance of humans and non-human primate species in tasks that involve social learning, researchers provide participants with varying amounts of information about how to solve the task. End-state conditions show a “solved” problem or apparatus^4–7^; ghost conditions provide some information about object movement or other causal relation^7–14^; and full social demonstrations show an agent’s body movements as well as changes to an apparatus^4–16^. If the outcome achieved by the social demonstrator is repeated at a higher rate than outcomes revealed or shown in some other way, researchers usually conclude that social information has been given priority over other sources. However, there may be reasons that doing what another individual does or has done is in some way rewarding (regardless of whether an actual reward has been observed), and therefore more worth reproducing than an outcome presented in a different way. For example, the presence of a conspecific might draw an observer’s attention to part of an apparatus, with learning occurring as a byproduct of that attentional focus. Or an observer might have prior experience of reinforcement following the repetition of a conspecific’s actions, in which case their propensity to do something similar might be caused by generalisation of this learned association which would not apply in an individual learning context. This is because the natural behaviours performed by conspecifics are not random but rather constitute a subset drawn from the universe of possible behaviours; this subset is likely biased toward behaviours that have been previously rewarded with a payoff^17^.

In order to adequately address the question of how social and individual information are used, task demonstrations should provide unambiguous cues regarding what to do as well as what *not* to do. Furthermore, these cues should be consistent across information sources: the same unambiguous cue should provide equivalent information when acquired individually (i.e. through an individual’s own performance) or demonstrated socially. A task which flexibly provides information regarding either what should be repeated, or what should be avoided—in equivalent ways across sources—permits investigation of whether the source affects how these different types of information are responded to, relative to one another. This is a key question in understanding whether information from a social source is treated as if it had some kind of special (or simply different) status compared to information from other sources, which is independent of the inherent value of that information.

Tasks used to evaluate information use must also allow for fair comparisons between species. This can be difficult to achieve given that human children experience object-rich developmental contexts and generally have more opportunities to learn about affordances via object-based play than do non-human primates. In touchscreen tasks, stimuli can be varied such that each instance of a task is novel, and solutions do not rely on previously existing associations. This feature allows researchers to train participants to proficiency on a task, ensuring an understanding of a paradigm’s contingencies, and test them on novel instances of the task. The ability to train individuals to proficiency if required, and then present novel problems, allows for a fair(er) comparison between species.

**Figure 1:**
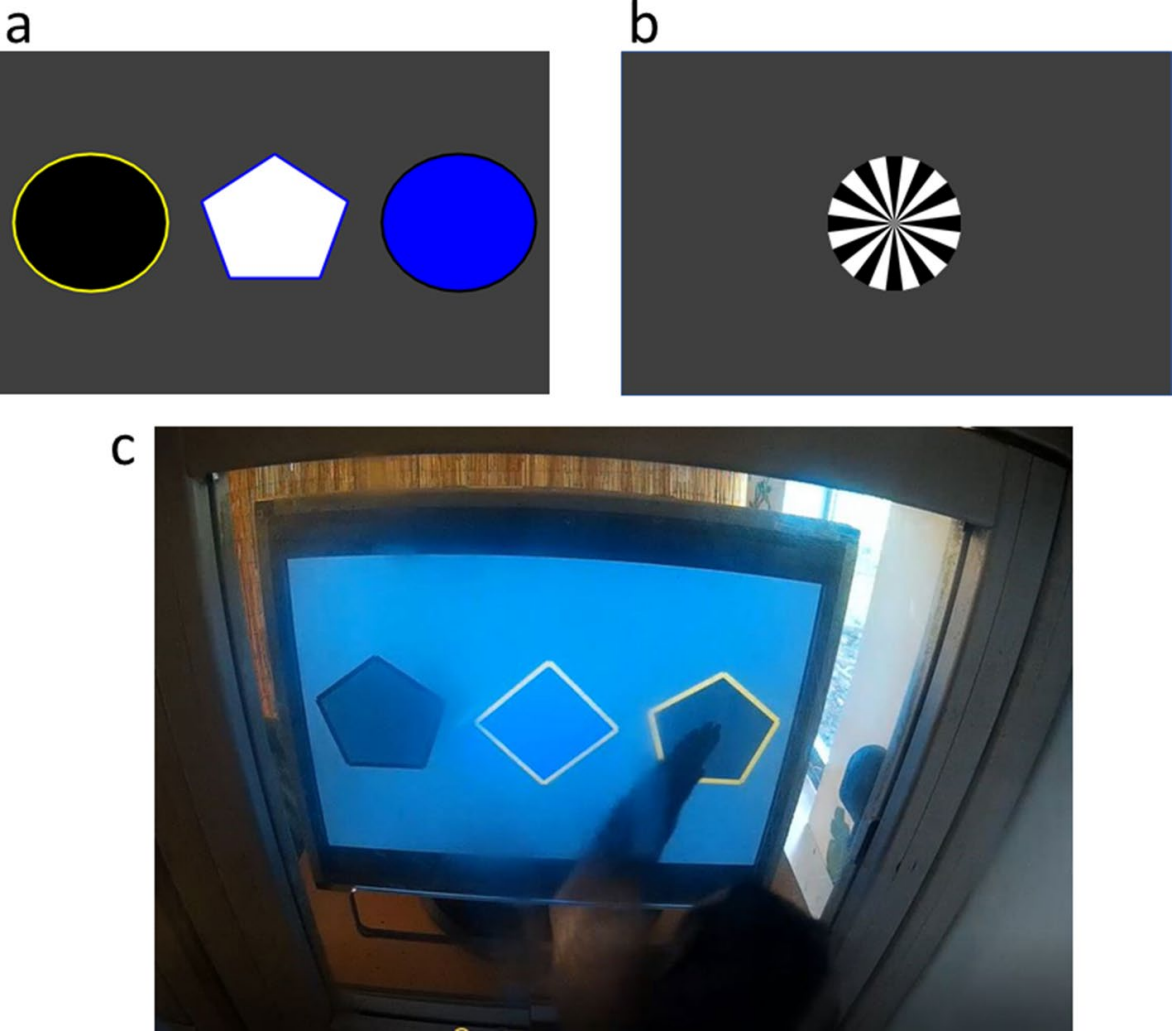
Example 3-stimulus problems. (**a**) At the beginning of an information trial (T1) or test trial (T2), stimuli are displayed on the screen. (If this represents a T1, the following test trial would look identical to this screen.) (**b**) If a rewarded stimulus is selected, a visual cue (sunburst) is displayed and an auditory click is produced as feedback. If a monkey selected the rewarded stimulus, a food reward is also dispensed; if a child selected the rewarded stimulus, they are allowed to add a sticker to their sticker sheet. If an unrewarded stimulus is selected (not pictured), the stimuli disappear and only the background is visible. (**c**) An individual monkey interacting with the apparatus. This is a different problem from that displayed in (**a**,**b**).

In this study, we designed a touchscreen task that allowed for equivalent information to be presented in social and individual conditions, as well as a third, animated cue, condition (Fig. 1). On an initial “information trial” (T1), a participant received some information about one of the stimuli: whether it was rewarded (“win”), resulting in a raisin or sticker, or unrewarded (“lose”), resulting in no reward. Individuals were therefore required to respond to the information available on each trial, instead of adopting an indiscriminate (or “blanket”) copying strategy^18–20^ or a blanket avoiding strategy^21,22^. Good performance could be achieved by reliably re-selecting rewarded stimuli and reliably avoiding unrewarded stimuli. Including both types of information in T1s therefore allowed us to determine whether information type differentially affected performance in the various source conditions. For example, we could evaluate whether there were differences in copying of unreinforced responses in the social and individual conditions.

We compared the task performance of 2- to 5-year-old children and capuchin monkeys. Capuchins, as social primates with large brains and an omnivorous diet^23,24^, are a useful comparison species with humans. Capuchins’ learning has been shown to be influenced by social processes^25,26^, and they can socially transmit behaviours in diffusion chain experiments^27^. While capuchin monkeys’ poor performance in tool-based tasks is surprising^28^, they can achieve good performance in computerised tasks operated by joystick^29–31^ or by touch^32^. Our previous research has shown that children in this age range understand the contingencies of tasks like this with no training^33^. However, we anticipated the need to train the monkeys on this task to ensure that they would treat the cues made available to them in T1 as information relevant to T2, whereas verbal scaffolding served to achieve this for the children (see “Methods” section).

The three information source conditions were individual, social, and animated cue. In the various conditions, the same information was available to the participant, but it was acquired by different means. In the individual condition, the participant selected one of the stimuli on T1 themselves. In the social condition, the adult human experimenter selected one of the stimuli. And in the animated cue condition, an animated hand appeared on the screen, and moved to make contact with (select) a stimulus before moving off the screen (see example video at https://osf.io/pwt65/). An information trial in the individual condition was therefore equally informative to an equivalent one in the social or animated cue condition.

The animated cue condition was included for several reasons. If a species difference in performance were to be found between the social and individual conditions, then the animated cue condition could be informative about the underlying mechanism. For example, if one group gives primacy to information that is the result of personal—individual—experience (e.g., participants have enhanced memory for their own responses, compared to anything experienced vicariously), then we would predict performance in the animated cue condition (along with the social condition) to be worse than that in the individual condition. And if one group responds to social information in special ways (e.g., they show a higher proportion of “repeat” or “stay” responses, even following unrewarded T1s, in the social condition^34^, as the result of a conformity-like response or attribution of knowledge or expertise to the social model), their performance in the animated cue condition (along with the individual condition) would be expected to show a different pattern. Therefore, this condition should provide information about the scope and type of difference in relation to the question of whether it is the social or the individual condition that is treated distinctively.

Finally, the size of the array allowed us to evaluate the fidelity of learning by comparing “problems” (particular T1–T2 pairings involving a specific set of stimuli) with different chance levels of performance. If a participant has learned (or correctly inferred) what the information in T1 means, they should repeat the selected stimulus at similar rates regardless of the number of distractor stimuli. If a participant simply has a bias towards repeating responses that have been associated with a reward (over those that have not), we would expect their rates of repeating to be influenced by the number of distractors. If only humans use the T1 information in a high-fidelity manner (repeating or avoiding stimuli at similar rates regardless of the number of distractors), this may help to explain why human cultures increase in complexity while non-human ones appear not to^10^. This is because, as solutions become increasingly less likely to be discovered through random exploration, the utility of (any kind of) information increases. If non-human primates use information in only a lower-fidelity manner, with rates of repetition or avoidance influenced by the number of distractors, this response pattern might not provide enough benefit to allow improbable but adaptive behaviours to persist, given the large number of alternative behaviours available in wild contexts. Results may therefore contribute to our understanding of why culture does (or does not) increase in complexity in various species.

## Results

Task performance was measured in two ways. One measure was whether the selection from T1 was repeated on the next trial: the “repeats” measure. This variable was binary, and took a value of 1 if the stimulus selected on T1 had been repeated in T2, and a value of 0 if it had not. This measure is useful when comparing responses on problems with different numbers of stimuli and therefore different chance levels of performance. The other measure was whether, on a given trial, an individual used a win-stay, lose-shift (WSLS) strategy. For example, if a participant saw a “win” in T1 and picked the same stimulus on T2, they had performed a win-stay; and if they saw a “lose” in T1 and picked a different stimulus on T2, they had performed a lose-shift (regardless of whether the shift had resulted in them finding the reward). This variable was also binary, and it took a value of 1 if a WSLS strategy had been used on a given trial, and a value of 0 if it had not.

Overall, the 29 children performed well on T2s but not at ceiling. They repeated the T1 selection after a win in 68% of 2-stimulus and 62% of 3-stimulus arrays, and repeated the T1 selection after a lose in 20% of 2-stimulus and 22% of 3-stimulus arrays. Of the 15 monkeys who participated in testing, 2 (Inti and Torres) showed mastery of the task contingencies, as evidenced by > 75% WSLS performance on the experimental task. The two task-proficient monkeys also performed well but not at ceiling; they repeated the T1 selection after a win in 93% of 2-stimulus and 87% of 3-stimulus arrays, and repeated the T1 selection after a lose in 35% of 2-stimulus and 31% of 3-stimulus arrays. The 13 non-task-proficient monkeys repeated the T1 selection after a win in 70% of 2-stimulus and 56% of 3-stimulus arrays, and repeated the T1 selection after a lose in 52% of 2-stimulus and 38% of 3-stimulus arrays. See Table 1 for full details of performance by trial type, and the Supplementary Information for additional analyses.

**Table 1:** Overall performance (percentage repeats) of children and capuchin monkeys in the various conditions.

Participant type	Percentage repeats											
	Individual condition				Social condition				Animated cue condition			
	2-stimulus win	2-stimulus lose	3-stimulus win	3-stimulus lose	2-stimulus win	2-stimulus lose	3-stimulus win	3-stimulus lose	2-stimulus win	2-stimulus lose	3-stimulus win	3-stimulus lose
Children (*N* = 29)	62.9	14.8	68.5	18.3	69.6	23.0	65.0	21.8	71.4	22.0	54.2	27.8
Monkeys overall	91.9	50.5	81.4	42.0	60.8	45.8	51.0	33.0	70.0	48.0	55.6	35.4
Inti	100.0	0.0	100.0	50.0	100.0	50.0	83.3	50.0	87.5	33.3	83.3	50.0
Torres	100.0	33.3	93.8	20.0	94.1	27.3	90.9	23.5	86.7	53.3	73.3	26.7
Non-task-proficient monkeys (*N* = 13)	90.6	58.9	77.3	44.2	51.3	48.1	43.0	34.2	65.5	48.1	50.0	35.6

Chance performance for the repeats measure is 50.0% in 2-stimulus trials and 33.3% in 3-stimulus trials.

### Task-proficient monkeys: effects of source, information type, and array size

**Figure 2:**
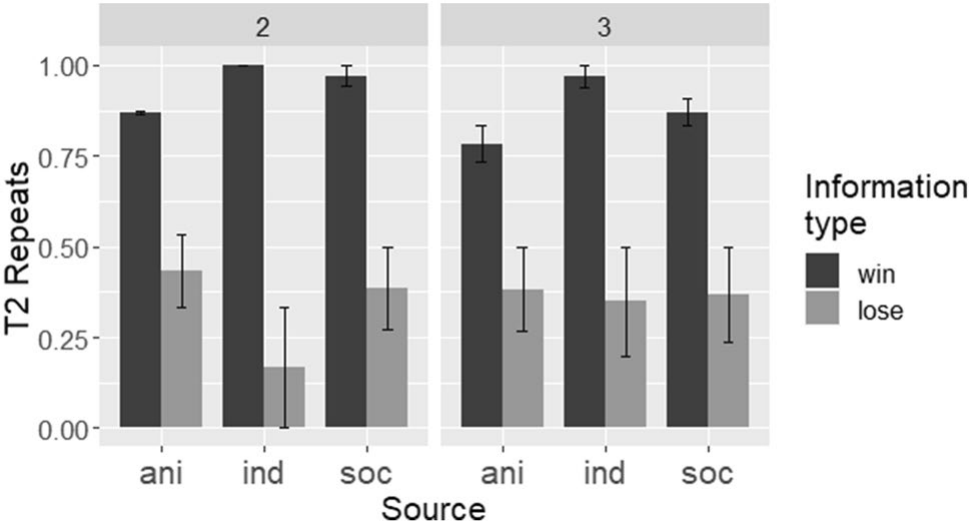
Task-proficient monkeys’ performance (as measured by T2 repeats), by source, information type, and array size. Bars show means of by-participant means, and error bars show bootstrapped 95% confidence intervals. *ani* animated cue, *ind* individual, *soc* social.

To examine task-proficient monkeys’ performance using the repeats measure (Fig. 2), we built a GLMM with logit link in *lme4*^35^, with a binary response variable of repeats; fixed effects of source (three levels: individual, social, and animated cue), information type in T1 (two levels: win and lose), array size (two levels: 2- and 3-stimulus arrays), and their interactions; and a random effect of participant ID. However, this model resulted in a singular fit. We next built an equivalent Markov chain Monte Carlo GLMM in a Bayesian framework using the *MCMCglmm* package^36^. This model had the same response variable and fixed and random effects as listed above; it had weak priors and was run with 100,000 iterations, a burn-in of 1000, and a thinning interval of 100. We assessed mixing and convergence with trace and posterior plots. This model showed a main effect of information type (more repeats after win [90%] than lose [33%] T1s; Estimate =  − 206.0, 95% CI = − 350.5 to − 59.9, effective sample size [ESS] = 825.2, *p*MCMC < 0.001). There was also an interaction between source and information type (Estimate =  − 263.6, 95% CI =  − 526.0 to − 28.6, ESS = 736.1, *p*MCMC = 0.034). Main effects of array size (*p*MCMC = 0.40) and source (*p*MCMCs ≥ 0.12) were not significant, and the credible intervals for these main effects encompassed 0; other interaction effects were also not significant, with credible intervals encompassing 0 (*p*MCMCs > 0.24).

To examine the source-information type interaction, we split the data set by information type. We created MCMCglmms with source as the only fixed effect, participant ID as a random effect, and the same parameters as those listed above. For lose T1s, the main effect of source was not significant (all 95% CIs encompassed 0, *p*MCMC ≥ 0.26). For win T1s, the main effect of source was significant, with more repeats after a win in the individual (97%) than the animated cue (82%) condition (Estimate = 2.73, 95% CI = 0.72 to 4.80, ESS = 53.4, *p*MCMC = 0.01).

### Children: effects of source, information type, and array size

**Figure 3:**
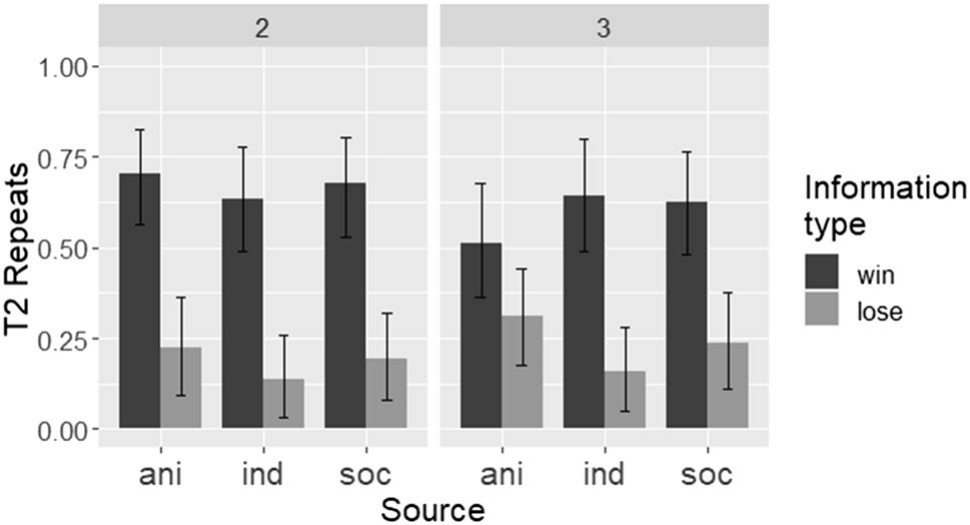
Children’s task performance (as measured by T2 repeats), by source, information type, and array size. Bars show means of by-participant means, and error bars show bootstrapped 95% confidence intervals. *ani* animated cue, *ind* individual, *soc* social.

To examine children’s performance using the repeats measure (Fig. 3), we built a GLMM with logit link in *lme4*, with a binary response variable of repeats, and fixed effects of source, information type, array size, and their interactions. A random intercept effect of participant ID and a by-participant random slope effect of session were included in the model. The main effect of source was not significant; full pairwise contrasts between all three source conditions using Tukey’s correction indicated that children did not differ in their repeating of the T1 selection between the individual and social (*b* =  − 0.53, SE = 0.45, Z =  − 1.2, *p* = 0.46), individual and animated cue (*b* =  − 0.23, SE = 0.44, Z =  − 0.53, *p* = 0.86), and social and animated cue (*b* = 0.30, SE = 0.48, Z = 0.63, *p* = 0.81) conditions. Information type had a significant effect, indicating more repeats after win than lose information trials (*b* =  − 2.5, SE = 0.48, Z =  − 5.3, *p* < 0.001). The main effect of array size was non-significant (*b* =  − 0.82, SE = 0.42, Z =  − 1.9, *p* = 0.054), but showed a trend toward fewer repeats in 3-stimulus arrays. The interaction between information type and array size was also marginally non-significant (*b* = 1.23, SE = 0.65, Z = 1.90, *p* = 0.058). Other 2-way and 3-way interactions were not significant (all *p*s > 0.08).

### Non-task-proficient monkeys: effects of source, information type, and array size

**Figure 4:**
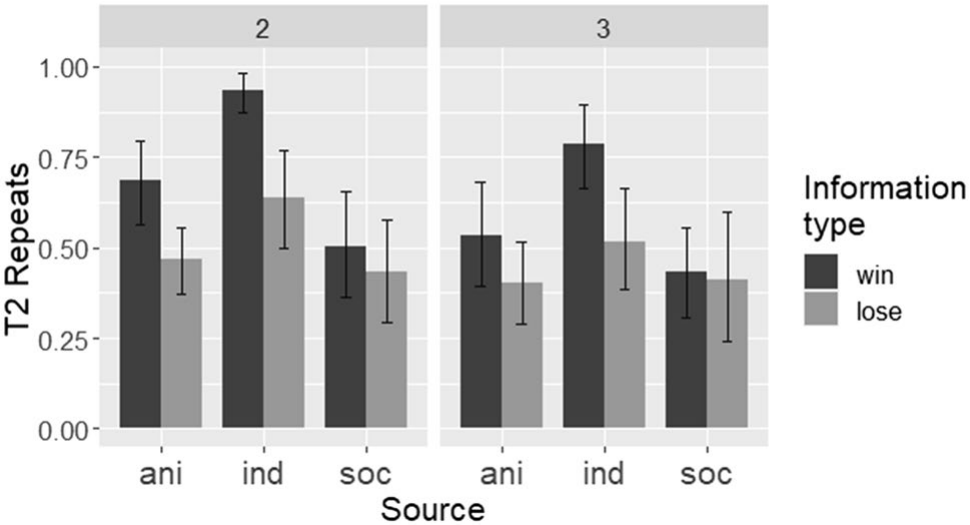
Non-task-proficient monkeys’ performance (as measured by T2 repeats), by source, information type, and array size. Bars show means of by-participant means, and error bars show bootstrapped 95% confidence intervals. *ani* animated cue, *ind* individual, *soc* social.

To examine non-task-proficient monkeys’ performance using the repeats measure (Fig. 4), we built a GLMM with logit link in *lme4*, with fixed effects of source, information type, array size, and their interactions. A random intercept effect of participant ID and a by-participant random slope effect of information type were included in the model. The main effects of source, information type, and array size were all significant. Full pairwise contrasts with Tukey’s correction for multiple comparisons showed that monkeys repeated more in the individual condition than in the social (*b* = 2.2, SE = 0.44, Z = 5.1, *p* < 0.001) and animated cue (*b* = 1.7, SE = 0.44, Z = 3.8, *p* < 0.001) conditions. The difference between the social and animated cue conditions was not significant (*b* =  − 0.55, SE = 0.33, Z =  − 1.7, *p* = 0.21). Like children and task-proficient monkeys, non-task-proficient monkeys repeated more after win than lose information trials (*b* =  − 0.77, SE = 0.34, Z =  − 2.3, *p* = 0.024). They also repeated more in 2-stimulus than 3-stimulus arrays (*b* =  − 0.66, SE = 0.33, Z =  − 2.0, *p* = 0.045), in line with the change in baseline probability of selecting a given stimulus. The interaction between source and information type was significant. A post hoc comparison using the *emmeans* package^37^ showed that the non-task-proficient monkeys repeated win selections more in the individual condition than the social (*b* =  − 1.9, SE = 0.29, Z =  − 6.6, *p* < 0.001) and animated cue (*b* = 1.5, SE = 0.29, Z = 5.1, *p* < 0.001) conditions; however, there was no such difference after lose trials (*p*s > 0.30).

### Additional analyses

We examined the effects of two other variables, full details of which are in the Supplementary Information. Briefly, task-proficient monkeys’ WSLS performance did not improve with post-training task experience (session number), while that of non-task-proficient monkeys actually decreased with post-training task experience. Children’s WSLS performance improved with task experience (session number). Additionally, child age (in months) was a positive predictor of WSLS performance.

## Discussion

Neither children nor task-proficient capuchin monkeys used social information in a distinctive way, as compared to other types of information. For children, there was no overall difference in performance—as measured by repeats—between the individual, social, and animated cue conditions. Researchers have previously found very good performance after social demonstrations of experimental tasks^8,10,15^. However, here we show that when the amount and quality of information are controlled for, the nature of the information—its derivation from an individual, social, or animated source—may not be the definitive factor. In this study, the lack of performance differences between the individual, social, and animated cue conditions, combined with the general pattern of staying after wins and shifting after loses, indicated that children were able to make use of the information they received regardless of source.

The task-proficient capuchin monkeys showed a performance pattern similar to that of the children, with more repeating after wins than loses and no main effect of source. However, their performance differed from children’s in the interaction of source and information type. These monkeys repeated more after a win in the individual condition than in the animated cue condition, with no difference between the sources after a lose. It is possible that the poorer performance in the animated cue condition could reflect a real disadvantage in using disembodied information, since it accords with previous findings of reduced learning from so-called “ghost” conditions (discussed further below). However, it should be noted that both of the task-proficient monkeys had been trained to proficiency in the individual condition, which could account for their better performance at test in this condition. Regardless of any difference between individual and animated cue conditions, task-proficient monkeys were able to learn equivalently well in the social and individual conditions. That is, it was not between the social and individual conditions that performance differences manifested (see Fig. 2).

Children and task-proficient capuchins repeated the demonstrated selection more after a win than a lose information trial. That is, both groups were sensitive to the type of information they received in T1. Despite the shared pattern of more repeats after wins, however, each group’s overall performance pattern exhibited distinctive characteristics in relation to information type. Specifically, children repeated the T1 selection less frequently than the capuchins. As we have previously reported^33^, and in accordance with others’ results^38^, children exhibit a “shift” bias in tasks like this. That is, while they are sensitive to the reward information in T1, they have a (weak) tendency to shift from the initially selected stimulus after both wins and loses, resulting in better performance after lose T1s. By contrast, the task-proficient monkeys here showed, if anything, a “repeat” or “stay” bias, meaning that overall, they repeated the T1 selection more frequently than the children. For example, in 2-stimulus problems after a win T1, children repeated on 68% of trials, compared to 93% for this same trial type for the task-proficient capuchins. After a lose T1 in 2-stimulus problems, children repeated on 20% of T2s, while task-proficient monkeys repeated 35% of the time. We previously modelled children’s responses in a similar task; a model that incorporated a bias against repeating the T1 response better captured children’s actual performance than an unbiased model^33^. The task-proficient monkeys do not appear to have such a bias against repeating a previous response.

It is also noteworthy that non-task-proficient capuchin monkeys did not perform like the task-proficient monkeys or the children in regard to source and information type. While they too were sensitive to the type of information provided in T1, repeating more after wins than loses, this difference was fairly small in the social and animated cue conditions. These monkeys also repeated more in the individual condition than in the other conditions; and they tended to repeat more after wins in the individual condition than in the other conditions. Perhaps this is due to the salience of receiving both direct (a raisin) and indirect reinforcement after a win in the individual condition. The salience of the feedback after win T1s in the social and animated cue conditions (visual and auditory cues, but no food, and experienced vicariously, rather than self-generated) may have been lower, leading to fewer repeats. While the performance of the task-proficient monkeys showed that some members of this species were able to learn the significance of the information in T1 (and perform well regardless of source), the performance of the non-task-proficient monkeys indicated that they did not make as clear a distinction between win and lose T1s, and that there was differential sensitivity to this difference depending on the information source.

The non-task-proficient monkeys’ higher rates of repetition in the individual condition may hint at an alternative perspective on effects sometimes interpreted as showing that non-human primates exhibit relatively limited proclivity for social learning compared with humans. For example, evidence of limited observational learning in tasks that primates are nonetheless capable of mastering through individual trial-and-error (e.g.^39^) is perhaps better understood as consequences of the subjects’ failure to understand the relevance of the task feedback (whether directly or vicariously experienced) as a guide to future action. This makes repetition of successful responses far more likely under individual learning as a result of simple reinforcement, as this requires no such insight, in contrast to social information use. This interpretation is bolstered by the contrasting pattern of performance in the task-proficient monkeys, who clearly *did* understand the relevance of the task cues, and showed limited effects of source, exhibiting a pattern of performance more similar to the children. If this interpretation is correct, apparent human advantages for social learning may reflect an understanding of the relevance of information regardless of source, in combination with a non-human advantage for learning from direct experience. We return to this point at the end of the Discussion.

The effect of array size requires careful interpretation, given the differing levels of chance performance between 2- and 3-stimulus arrays. Generally, non-task-proficient monkeys’ performance accorded with what would be expected based on chance levels and associative learning. That is, they repeated after wins more often with 2-stimulus (70%) than 3-stimulus (56%) arrays (reflecting the change in chance probabilities from 50 to 33%), in line with a lower expectation of repeats when more stimuli are available if deviations from ceiling performance are simply due to noise. They repeated after lose T1s more often in 2-stimulus (52%) than 3-stimulus (38%) arrays, in line with the same principle. The performance of task-proficient monkeys and children was not affected by chance probabilities to nearly the same extent. This seems consistent with our interpretation that both the children and task-proficient monkeys recognised the predictive relevance of the task cues, in a way that the non-task-proficient monkeys did not (despite some differential responding to win/lose cues). Below-ceiling performance in these groups is therefore more likely to be attributable to competing biases or preferences (such as a motivation for exploration over exploitation in the children^40^) rather than simple noise.

Had we found differences between the individual and social conditions, the animated cue condition would have assisted in examining the possible underlying mechanisms. The finding of non-differentially effective information use from an animated cue condition and a social condition by task-proficient monkeys and children is unusual, given that a number of previous studies have shown impoverished learning from ghost conditions^8–13,41–43^, but see^14^. It is possible that the difference between this study and the others is due to participants engaging in multiple trials, with corresponding opportunities to learn the game’s strategy. Children and monkeys might therefore have achieved better performance in this particular animated cue condition than in a one-off physical task. Another possible reason for the differing results is the simple, straightforward contingencies of our task. Children and monkeys did not need to learn complex object relationships or sequences to select an item that was (likely to be) rewarded, and were therefore able to easily utilise the information presented to them, regardless of its source. Finally, the selection of shapes in this task did not require fine motor control, meaning that individuals’ performance would not have been limited by the need to both create and execute a matching motor plan (the correspondence problem), as three-dimensional tasks might require.

The following limitations should be taken into consideration when interpreting the results. First, the task was abstract, with limited ecological relevance to members of both species. Second, only 2 of 15 monkeys passed the performance criterion; it is possible that there was something special or different about these monkeys, and their performance may not generalise widely. Third, the social model—a human demonstrator for both species—may have had differential relevance for the two groups. However, if the status of the model as a conspecific (for the children) or heterospecific (for the monkeys) influenced performance, we would expect to see differences in the response to source conditions between the children and the task-proficient monkeys. Because each group treated social information similarly to both individual and animated cue information, the model’s status appears to have had minimal influence on the outcome.

How do our results relate to existing knowledge on social learning and cultural evolution in humans and non-human primates? Children have previously been found to have an apparent performance advantage over non-human primates in tasks involving the use of social information^44^. In these tasks, children often use social information with ease, while non-human primates do not. However, given the results of the present study, this may not be due to a specialised adaptation for social information use among humans. Instead, it could be due to other factors, such as increased attention to social information, possibly as a result of developmental experiences^45^, or a more general metacognitive ability to recognise the usefulness or significance of a variety of types of information, including that arising from social sources. If humans are able to recognise the utility of various types of information from the world around them, this may help to explain the apparent species uniqueness of cumulative cultural evolution. In contrast, for non-human primates, associative cues may be more salient when experienced directly in response to an individual’s own activity rather than vicariously, as was the case for the non-task-proficient monkeys in this study, and to some extent the task-proficient monkeys as well.

In summary, in simple tasks like the one presented here, children and task-proficient capuchin monkeys do not seem to use social information in a unique way. Indeed, if anything, the task-proficient monkeys engaged in more copying after witnessing wins, across all conditions, than the children did. This result seems to indicate that, when the value of information is controlled for across conditions, the source of information is not the most important determinant of the future use of that information. We do not know whether this result would generalise to a more complex task which required greater use of cognitive resources. Such a question should be the topic of future research, which could usefully provide insight into species differences in information use on complex tasks.

## Methods

### Monkeys

#### Participants

We tested capuchin monkeys (*Sapajus apella*) at the Living Links to Human Evolution Research Centre at Edinburgh Zoo^46^. At the time of this research, 35 capuchin monkeys in two social groups, East and West, were housed at the facility. These monkeys had previously been trained via positive reinforcement to enter the research cubicles, allow themselves to be temporarily separated from their group, and participate in cognitive testing. When separated from the group, a monkey could request to re-join its social group by touching the sliding door of the cubicle; experimenters opened the door to accommodate this, regardless of the point in a session in which this occurred. Research was therefore voluntary, and not every monkey participated. Details of the 17 monkeys who did participate in task training or testing are given in Supplementary Information Table S1. A further 4 monkeys participated in training to use the touchscreen but not frequently enough to progress to task training; and 14 monkeys never participated in any training or testing.

#### Ethical approval

Ethical approval for the primate research was granted by the Animal Welfare and Ethical Review Body at the University of Stirling, under application number AWERB (17/18) 025. Animals were not food or water deprived. Research was conducted in line with the guidance provided by The Association for the Study of Animal Behaviour (2017) in *Guidelines for the treatment of animals in behavioural research and teaching*^47^.

#### Apparatus

To display the training and experimental tasks, we used a touch-sensitive monitor (Elo 1939L; 37.5 cm [width] × 30 cm [height]) controlled via a tablet (Microsoft Surface). The custom programme for presenting the task was written in PsychoPy^48^. The screen and tablet were placed on a mobile trolley for ease of movement. Monkeys interacted with the touchscreen through a front-facing cubicle window with a horizontal opening (see Fig. 1c).

#### Procedure

We planned to train monkeys on two training tasks, the first of which presented two stimuli in every problem and the second of which presented three. Once monkeys passed a performance criterion on both the 2-stimulus and 3-stimulus training tasks, they would progress to the experimental task. Our goal was to train and test as many monkeys as possible in the time period allotted; we thought it likely, however, that not all monkeys would pass all training levels. Additional details of the procedure are available in the Supplementary Information.

##### Training tasks

We previously trained the capuchin monkeys on a 2-stimulus task paradigm in one of two information source conditions (social or individual information only in all information trials^49^). In this training task, monkeys were presented with problems that showed two stimuli on a background. Stimuli were shapes with a randomly generated number of sides and randomly assigned fill and outline colours, given the limits of platyrrhine colour vision^50^. In the first trial (T1) of a training problem—the information trial—a monkey either saw the experimenter select a stimulus (social condition) or selected a stimulus themselves (individual condition). The monkey was then allowed to select a stimulus on the four subsequent trials of that problem (T2 through T5). After completing all five trials of a problem, they were presented five trials of the next problem, and so on. Each session had four problems, for a total of 20 trials per session. We focused our analyses on the second trial (T2) of each problem; the remaining trials in a problem were to reinforce learning of the task structure.

For half of the problems in a training session, the initial selection on the information trial was randomly assigned to be rewarded; for the other half, unrewarded. This was regardless of the identity or position of the stimulus, in order to prevent monkeys from developing side biases.

When the experimenter or monkey selected a rewarded stimulus, both stimuli disappeared, a sunburst visual cue appeared in place of the rewarded stimulus, and an auditory click was generated by the programme. Additionally, when monkeys selected a rewarded stimulus, they received a raisin. When an unrewarded stimulus was selected, all stimuli disappeared, a time-out screen showing only the background colour was displayed for 3 s, and no raisin was dispensed. For all trials in a given problem, the same stimulus, in the same location, was the rewarded stimulus. The purpose was to train the monkeys to recognise the predictive value of the cue revealed in T1 for the corresponding T2.

We established a performance criterion on the 2-stimulus training task for monkeys to progress to the 3-stimulus training task. The criterion was ≥ 75% use of WSLS behaviour on the T2s in a session, in addition to performance (successful location of the rewarded stimulus) of ≥ 75% on T3 through T5 in that session, for three consecutive sessions. This is similar to performance criteria used in previous research^51^.

The 3-stimulus training task was similar to the 2-stimulus task, but the number of stimuli was increased to 3 (see Fig. 1a). The 3-stimulus training continued for each monkey in the same condition (social or individual) as their 2-stimulus training. With 3 stimuli, a win on T1 was more informative about the location of the reward than a lose.

The criteria to progress from the 3-stimulus training task to the experimental task were similar to those for progressing from 2-stimulus training: ≥ 75% use of WSLS on the T2s in a session, as well as ≥ 75% success on T3 through T5, for three consecutive sessions. Note that this does not require *finding* the reward on T2 after a lose T1, but rather use of the strategy that *could* result in finding it. Some monkeys met this criterion; however, it became apparent that few additional monkeys would ultimately pass the 3-stimulus training criteria in the time allotted for this study. Therefore, after several months of testing, we allowed all monkeys who had participated in any 2-stimulus training sessions to progress to the experimental task. This enabled us to collect data from a larger number of monkeys, and to compare the performance of monkeys that had shown relative proficiency in training to that of those who had shown limited proficiency.

While meeting criterion on both training tasks was necessary for a monkey’s data to be analysed with the task-proficient group, it was not sufficient. For this we established an additional performance criterion, of continued > 75% WSLS performance on the experimental task. For additional details about this, see the Supplementary Information.

##### Experimental task

In the experimental task, as in training, a single session consisted of four different problems, with five trials per problem. Half of the four problems contained a 2-stimulus array, and half contained a 3-stimulus array. T1s were performed by the same source (individual, social, animated cue) within each session; and the source differed between sessions in a pseudorandom order. In the individual condition, the monkey selected a stimulus on T1; in the social condition, the experimenter selected a stimulus; and in the animated cue condition, an animated hand selected a stimulus. Half of T1s were rewarded (wins), and half were unrewarded (loses). The number of sessions monkeys completed depended on how frequently they entered the testing cubicles (range, 3 to 43).

### Children

#### Participants

We tested children (*N* = 32; age 33 to 61 months; 17 female) at a University-based kindergarten in the United Kingdom. Data from three participants were excluded due to their participation in only two sessions, leaving a final sample of 29 children (15 female); three 2-year-olds, nineteen 3-year-olds, five 4-year-olds, and two 5-year-olds.

#### Ethical approval

Ethical approval for the child research was granted by the General University Ethics Panel at the University of Stirling, under application number GUEP467, and by the Stirling Psychology Ethics Committee. Research was conducted in accordance with the British Psychological Society *Code of Human Research Ethics*^52^.

When parents enrol their child in this kindergarten, they provide written informed consent for the child to participate in research conducted on-site. Parents are also given the opportunity to opt their child out of individual studies for any reason. For 2 weeks before data collection began, a notice with details of the research project and opt-out forms were placed on the kindergarten notice board. Any child whose parent(s) opted them out of participation was not asked to participate. Before testing, the experimenter sought each child’s assent to participate in a game; testing commenced only after the child agreed to play the game.

#### Apparatus

To display stimuli and record children’s responses, we used a touch-sensitive tablet with capacitive touch technology (23 cm [width] × 15 cm [height]). The custom programme used to present the task was written in PsychoPy^48^.

#### Procedure

Each child was tested in three sessions on three separate days; testing sessions were at least 4 days apart. Each session used a different information source (individual, social, or animated cue), and the order of sources was counterbalanced across participants. In each session, a child was presented with 8 problems; these problems contained a random mix of win and lose information trials (50% each) and 2- and 3-stimulus arrays (50% each). Each problem had two trials: the information trial (T1) and a single test trial (T2).

Each child was given a sheet of paper with a picture of a cartoon dog. The experimenter told the child that in the game they were about to play, they would be looking for a ball (the sunburst), which was hiding. If the child found the ball, they could collect stickers on the sheet; the dog would like them to find as many balls as they could. For the paired trials, the first trial consisted of “taking a peek” and the second trial consisted of looking for the ball. For example, in the social condition, the experimenter said, “It’s my turn to take a peek first, and then you can look for the ball”. In the individual condition, the experimenter said, “It’s your turn to have a peek, and then you can look for the ball”. In the virtual condition, they said “Let’s take a peek first, and then you can look for the ball”.

After each information trial, the child performed the corresponding test trial (T2). On T2, if a child selected the rewarded item (“found the ball”), they were allowed to select a small sticker and place it onto their sticker sheet. If they did not select the rewarded item, the experimenter said, “Oh no, no ball! That’s OK, let’s do another one,” and the game continued with the next problem.

### Statistical analyses

We analysed the data using generalised linear mixed models (GLMMs) in the *lme4*^35^ and *MCMCglmm*^36^ packages in R version 3.6.3^53^. Pairwise comparisons of main effects with more than two levels were done with the *multcomp* package^54^.

## Supplementary Information

Supplementary Information.

## Data Availability

Data are available at https://osf.io/pwt65/.
