# Supplementary material for: The use of individual, social, and animated cue information by capuchin monkeys and children in a touchscreen task
Source: Sci Rep. 2021 Jan 13;11:1043. doi: 10.1038/s41598-020-80221-4 (PMC7806602; doi:10.1038/s41598-020-80221-4)
Supplement: Supplementary file 1 — Supplementary Information. [file 41598_2020_80221_MOESM1_ESM.docx]

**Supplementary Information for**

**The use of individual, social, and animated cue information by capuchin monkeys and children in a touchscreen task**

*Elizabeth Renner, Donna Kean, Mark Atkinson, and Christine A. Caldwell*

**Table S1.** Capuchin monkey (*Sapajus apella*) characteristics and training and experimental details

| **Name** | **Sex** | **Group** | **Age in years (as of the beginning of testing in November 2017)** | **Training condition** | **Met criterion on 2-stimulus training? (total no. sessions)^a^** | **Met criterion on 3-stimulus training? (total no. sessions)^a, b^** | **Total no. of experimental sessions (no. in individual, social, virtual)** |
| --- | --- | --- | --- | --- | --- | --- | --- |
| Alba | F | West | 6 | Individual | Y (15) | Y (16) | 24 (8, 8, 8) |
| Anita | F | East | 20 | Social | N (10) | - | 3 (1, 1, 1) |
| Bear | M | West | 4 | Individual | N (4) | - | 8 (3, 2, 3) |
| Carlos | M | East | 11 | Social | N (40) | - | 18 (6, 6, 6) |
| Chico | M | East | 8 | Social | N (39) | - | 28 (9, 9, 10) |
| Flojo | M | East | 6 | Individual | Y (26) | N (6) | 0 |
| Hazel | F | West | 4 | Individual | N (47) | - | 17 (6, 5, 6) |
| Inti | M | West | 8 | Social | Y (22) | Y (13)  Plus 33 refresher sessions | 16 (5, 5, 6) |
| Junon | F | East | 17 | Individual | Y (28) | Y (7) | 43 (14, 14, 15) |
| Kato | M | East | 12 | Social | Y (13) | N (49) | 17 (6, 5, 6) |
| Luna | F | West | 6 | Social | Y (7)  Plus 23 refresher sessions | - | 20 (6, 7, 7) |
| Mekoe | M | West | 9 | Social | N (7) | - | 12 (4, 4, 4) |
| Pedra | F | West | 9 | Social | N (7) | - | 0 |
| Pixie | F | West | 4 | Social | Y (9)  Plus 21 refresher sessions | - | 12 (4, 4, 4) |
| Ruben | M | East | 7 | Individual | N (46) | - | 25 (8, 9, 8) |
| Torres | M | West | 6 | Individual | Y (17) | Y (4)  Plus 11 refresher sessions | 43 (14, 14, 15) |
| Ximo | M | West | 7 | Individual | Y (16) | Y (16) | 21 (7, 7, 7) |

^a^See the text for details about refresher sessions.

^b^A dash indicates that a monkey never participated in 3-stimulus training.

**Methods**

*Apparatus*

A digital camera was used to record video of experimental sessions with monkeys. Sessions with children were not video recorded.

*Procedure: monkeys*

We provide here additional information on how monkeys were determined to be task-proficient, and details of refresher sessions.

Proficiency. We initially established meeting criterion on the 3-stimulus training task as a benchmark that we thought would ensure monkeys understood the task and could use the T1 information as information. However, upon transfer to the experimental task, some of the monkeys who had met criterion on 3-stimulus training did not perform as if they understood the relevance of the T1 information. For reasons outlined in the main text Introduction (regarding the need for a task that involves an unambiguous cue indicating whether a response should be repeated or avoided), our ability to make the intended comparisons across source conditions depended on the monkeys achieving a level of performance that gave confidence in their appreciation of the predictive relationship between T1 and T2. This also needed to encompass performance above chance level for information about both rewarded and unrewarded stimuli. As a result of this, we could not justify including lower-performing monkeys in the sample of “task-proficient” monkeys. We therefore established an additional performance criterion for monkeys to be included in the task-proficient group. This additional criterion was overall T2 WSLS performance on the experimental task of >75%. The result was that three monkeys (Alba, Junon, and Ximo) who had met the 3-stimulus training criterion were not included in the task-proficient group, while two monkeys (Inti and Torres) who met the 3-stimulus training criterion remained in the task-proficient group. The highest-performing monkey who was not included in the task-proficient group (Alba) used WSLS on 67% of T2s overall. We believe this endorses our decision not to group these individuals within the task-proficient category, as they fell considerably short of the 75% performance level we had set both as an a priori training criterion and as a post-hoc inclusion criterion. See Results below for additional support for this requirement regarding proficiency levels.

Refresher sessions. During the course of data collection, pauses in testing occurred between sessions. One was to assess monkeys’ training progress and evaluate how to proceed; another was to design and obtain institutional approval to carry out the experimental task. It was decided that, to progress to the next level, monkeys’ criterion-meeting sessions must have occurred in the preceding 2 weeks. Therefore, after a pause, monkeys were given “refresher” sessions, and would need to meet criterion in the refresher sessions in order to progress to the next phase of the experiment. The following monkeys who passed criterion in one training phase received refresher sessions. Two monkeys (Luna and Pixie) met criterion on 2-stimulus training in fairly few sessions (7 and 9, respectively); their training was paused for 1 month, to allow other monkeys to catch up with them so that multiple individuals could progress around the same time, and then resumed. During the refresher sessions, they did not meet criterion on 2-stimulus training again. Therefore, we continued 2-stimulus training sessions with them, and they were ultimately included with the group of monkeys who were subsequently moved on to the experimental task without meeting criterion on 3-stimulus training.

Inti met criterion on 3-stimulus training in 13 sessions, and was given refresher sessions to allow the rest of the monkeys to catch up. He did not meet the 3-stimulus training criterion again when given refresher sessions, and was moved on to the experimental task with the other monkeys who had not met criterion. Torres met criterion on 3-stimulus training in 17 sessions; because this was shortly before the second pause, he was given additional refresher sessions until the end of that phase. He then met criterion in 3 refresher sessions when testing resumed.

*Procedure: children*

Previous research [1] indicates that children spontaneously understand the relevance of the information trial, and respond in ways that suggest they infer the predictive relationship between T1 and subsequent trials, from early in the experiment. Therefore, we did not do any training with the children before the experiment began.

For five participants, computer errors occurred during testing, which involved the programme exiting before all 8 problems in a session had been completed. When this happened, the experimenter re-started the programme, and the child completed 4 to 8 additional problems. Only the first 8 problems completed by the child were used in data analysis; any further problems were not analysed.

**Results**

*Proficiency groups*

To confirm that the partitioning of the monkey groups (into task-proficient and non-task-proficient monkeys) was supported by the data, we compared the task performance of the monkeys in the task-proficient group (*N* = 2), monkeys who passed 3-stimulus training but whose subsequent performance in the experimental task was poor (“passing”; *N* = 3), and monkeys who never passed 3-stimulus training (non-task-proficient monkeys; *N* = 10). We used a logit link GLMM with WSLS as the response variable (binary), monkeys’ performance group (three levels: task-proficient, passing, and non-task-proficient) as a fixed effect, and participant as a random effect. The effect of performance group was significant. Pairwise comparisons (with Tukey’s correction for multiple comparisons) indicated that task-proficient monkeys’ performance was significantly better than that of both passing monkeys (*b* = 0.87, SE = 0.22, Z = 4.0, *p* < 0.001) and non-task-proficient monkeys (*b* = 0.90, SE = 0.19, Z = 4.6, *p* < 0.001). The difference between passing and non-task-proficient monkeys was not significant (*b* = 0.02, SE = 0.16, Z = 0.15, *p* = 0.99).

*Effect of experience*

We assessed whether experimental task experience (using a proxy of session number) affected various groups’ performance in the task. For non-task-proficient monkeys, we used a GLMM with logit link, with T2 WSLS as the (binary) response variable, session number (centred) as a fixed effect, and participant as a random effect. The main effect of session number was significant, with a negative estimate (*b* = −0.018, SE = 0.008, Z = −2.32, *p* = 0.02). This indicates that on the whole, non-task-proficient monkeys’ performance actually declined slightly with post-training task experience.

For task-proficient monkeys, we built a GLMM with logit link, with T2 WSLS as the (binary) response variable, session number (centred) as a fixed effect, and participant as a random effect. This model had a singular fit. Therefore, we examined the potential effect of experience separately for Inti and Torres, using GLMs. Each model had T2 WSLS as the response variable and session number (non-centred) as the fixed effect. For each of these monkeys, the main effect of session number was not significant (*p*s > 0.28), indicating that task-proficient monkeys’ post-training task performance did not change with experience.

For children, we used a GLMM with logit link, with T2 WSLS as the (binary) response variable, session number (non-centred) as a fixed effect, and participant as a random effect. Session had a significant positive effect on WSLS performance (*b* = 0.40, SE = 0.11, Z = 3.7, *p* < 0.001), indicating that children’s use of WSLS increased with experience in the task.

*Effect of child age*

Although this was not one of our hypotheses, we thought it likely that some children might have been more task-proficient than others, and that age could be a significant predictor of any difference. We therefore used a GLMM with logit link to examine the effect of age on WSLS performance, with a binary response variable of WSLS, a fixed effect of age in months (centred), a random intercept effect of participant, and a random by-participant slope effect of session. This model showed that age had a significant positive effect on WSLS (*b* = 0.034, SE = 0.017, Z = 2.0, *p* = 0.046).

**Discussion**

While neither group of monkeys improved their performance across sessions, children did. This was true even though most monkeys took part in more sessions (range: 3 to 43) than the children, who took part in three sessions each. Because children did not receive training prior to the task, any learning they did occurred while they were taking part in the task. Although children’s performance improved with session number (from a mean of 66% WSLS in the first session to 81% in the third), it seems clear that they grasped the contingencies of the task from early in the experiment, with performance exceeding average capuchin performance from the first session.

**References**

1. Atkinson, M., et al. Robust, source-independent biases in children’s use of socially and individually acquired information. *J. Exp. Psychol. Gen.*; 10.1037/xge0000959 (2020).
